# Smartphone Apps to Support Falls Rehabilitation Exercise: App Development and Usability and Acceptability Study

**DOI:** 10.2196/15460

**Published:** 2020-09-28

**Authors:** Helen Hawley-Hague, Carlo Tacconi, Sabato Mellone, Ellen Martinez, Claire Ford, Lorenzo Chiari, Jorunn Helbostad, Chris Todd

**Affiliations:** 1 University of Manchester Manchester United Kingdom; 2 Manchester Academic Health Science Centre, Manchester Manchester United Kingdom; 3 Health Sciences and Technologies-Interdepartmental Center for Industrial Research University of Bologna Bologna Italy; 4 mHealth Technologies s.r.l Bologna Italy; 5 Department of Electrical, Electronic and Information Engineering "Guglielmo Marconi" University of Bologna Bologna Italy; 6 Manchester University NHS Foundation Trust Manchester United Kingdom; 7 Norwegian University of Science and Technology Trondheim Norway; 8 NIHR Applied Research Collaboration Greater Manchester Manchester United Kingdom

**Keywords:** aged, postural balance, telerehabilitation, patient compliance, accidental falls

## Abstract

**Background:**

Falls have implications for older adults’ health and well-being. Strength and balance interventions significantly reduce the risk of falls. However, patients do not always perform the unsupervised home exercise needed for fall reduction.

**Objective:**

This study aims to develop motivational smartphone apps co-designed with health professionals and older adults to support patients to perform exercise proven to aid fall reduction and to explore the apps’ usability and acceptability with both health professionals and patients.

**Methods:**

There were 3 phases of app development that included analysis, design, and implementation. For analysis, we examined the literature to establish key app components and had a consultation with 12 older adults attending a strength and balance class, exercise instructors, and 3 fall services. For design, we created prototype apps and conducted 2 patient and public involvement workshops, one with 5 health professionals and the second with 8 older adults from an exercise group. The apps were revised based on the feedback. For implementation, we tested them with one fall service and their patients for 3 weeks. Participatory evaluation was used through testing, semistructured interviews, and focus groups to explore acceptability and usability. Focus groups were conducted with the service that tested the apps and two other services. Qualitative data were analyzed using the framework approach.

**Results:**

On the basis of findings from the literature and consultations in the analysis phase, we selected Behavior Change Techniques, such as goal setting, action planning, and feedback on behavior, to be key parts of the app. We developed goals using familiar icons for patients to select and add while self-reporting exercise and decided to develop 2 apps, one for patients (My Activity Programme) and one for health professionals (Motivate Me). This enabled health professionals to guide patients through the goal-setting process, making it more accessible to nontechnology users. Storyboards were created during the design phase, leading to prototypes of “Motivate Me” and “My Activity Programme.” Key changes from the workshops included being able to add more details about the patients’ exercise program and a wider selection of goals within “Motivate Me.” The overall app design was acceptable to health professionals and older adults. In total, 7 patients and 3 health professionals participated in testing in the implementation phase, with interviews conducted with 6 patients and focus groups, with 3 teams (11 health professionals). Barriers, facilitators, and further functionality were identified for both apps, with 2 cross-cutting themes around phone usability and confidence.

**Conclusions:**

The motivational apps were found to be acceptable for older adults taking part in the design stage and patients and health professionals testing the apps in a clinical setting. User-led design is important to ensure that the apps are usable and acceptable.

## Introduction

### Background

With a rise in the ageing population, there is an increase in the number of falls [1], and around a third of people aged 65 years and above fall each year [1]. Falls have major implications for the health and well-being of older adults. Evidence shows that strength and balance interventions can significantly reduce the risk of falls, rate of falls, and fall injuries [2-5]. However, to be effective, older adults need to reach an adequate level of exercise (thrice a week) and maintain it over time [3]. New innovative digital solutions that support the maintenance of fall prevention exercises and thereby reduce the risk of falls and re-referral to services are needed [6].

The Otago [7] home-based and Falls Management Exercise (FaME) group–based [8] programs are the two main cost-effective strength and balance programs delivered in the United Kingdom [9,10]. However, implementation of these programs by health services often does not conform with evidence-based protocols [9]. Fall rehabilitation is often only delivered over a short period of time, and contact is normally once a week [9]. Patients do not perform the required (unsupervised) home exercises needed to maintain the adequate levels of strength and balance [11] and have poor adherence to the intervention after discharge from rehabilitation services [12,13].

There is emerging evidence supporting the use of mobile phone–based healthy lifestyle programs [14,15], such as those that help increase physical activity [16,17]. King et al [18] developed and tested mobile apps based on the behavior change theory designed to motivate adults aged 45 years and older. One of these included personalized goal setting and behavioral feedback, receiving positive feedback from participants and increasing physical activity. Evidence suggests that mobile phones are more usable than other devices [19]. The proportion of older adults using smartphones is growing rapidly, with 39% of those aged 65 to 74 years and 15% of those aged above 75 years found to be using smartphones [20]. The advantages of smartphones over tablet devices is that the person is more likely to carry it with them and to leave it switched on when not directly using it, which can ensure the delivery of feedback in real time.

If we can use technology to support older adults to maintain an adequate level of strength and balance training, we could support them to maintain health and independence, reduce their fall risk, and prevent re-access to rehabilitation and hospital admissions. Previous research suggests that technologies that support fall prevention are acceptable to older adults as long as they are simple, reliable, effective, and tailored to individual needs [21]. A majority of smartphone apps used for fall prevention focus on risk assessment, rather than support for fall prevention exercise [22]. There is an ongoing trial of a smartphone-based strength and balance app, but this focuses solely on balance exercises [23] and is used on tablet devices. Another app that has been developed in Sweden is designed to be used alone by older adults, rather than in the management of falls as part of rehabilitation with a health professional [24].

### Theoretical Approaches

Previous studies have shown that attitudes and beliefs are important to uptake and adherence to exercise by older adults [25,26]. The theory of planned behavior (TPB) [27] is particularly useful for assessing older adults’ attitudes in relation to exercise [25,28]. The TPB is based on 3 core components:

1. Perceived behavioral control and perceived ease or difficulty of performing the behavior.
2. Social influences including subjective norms (beliefs of important people, eg, family), perceived social support (support from others for behavior), and modeling (following observed behavior of others).
3. Attitudes [27] focused on the advantages and disadvantages of the behavior (outcome expectations), and when related to adherence, whether these advantages have occurred.

There is evidence that interventions based on TPB can influence a person’s intent to exercise [28]. Adherence is also related to attitudes measured by the TPB [25,26].

In psychological literature, goal setting has been found to be a successful behavior change technique [29,30]. Other theories, such as the self-determination theory (SDT), can be related to goal setting and support the process of setting meaningful personal goals [31]. By choosing personal goals, we are more likely to satisfy intrinsic needs of relatedness (a sense of belonging), competence (a feeling of having the skills to achieve goals), and autonomy (that we can take direct action to make a change). From previous research exploring uptake and adherence to exercise programs by older adults, we know that goal setting, outcome-based feedback, and feedback designed to strengthen self-efficacy (eg, praise for progress so far) is particularly important for motivation to exercise [25,28,30,32]. It is important when describing interventions, particularly those based on behavioral theory, that we explore behavior change techniques (BCTs) utilized as mechanisms for this behavior change. The Behavior Change Taxonomy by Michie et al [33] has been developed to allow the BCTs used in an intervention to be clearly described and replicable.

Usability is an important part of designing successful technologies and has been described as the overall usefulness of a product and whether a person can use it for its intended purpose [34]. Acceptability is a multifaceted construct that reflects the extent to which people delivering or receiving a health care intervention consider it appropriate [35]. Smartphone apps are more likely to be acceptable to older adults and health care professionals if they are developed with them [36] using principles of human-centered design (HCD). The HCD ensures that the needs of the user are taken into consideration throughout the design process, and is a multi-stage process that allows for various iterations of a design to ensure it meets the needs of users [36]. Models such as the technology acceptance model (TAM), which focuses on whether a technology is perceived as useful and whether or not it is easy to use [37], are important when developing technologies. This model has also been expanded to include factors such as subjective norms and how this relates to perceived usefulness [38].

### Study Aims

The aims of our study are as follows:

1. To develop smartphone apps designed to support patients to exercise, based on psychological theory, and co-designed with health care professionals, older adults, and patients.
2. To explore whether two new smartphone-based apps designed to support adherence are usable and acceptable to health care professionals and patients when supporting a strength and balance home exercise program to prevent falls.

This study includes a range of interacting behavior change components and intervention development and is therefore based on the principles of the Medical Research Council framework for the development and evaluation of complex interventions [39].

## Methods

To achieve our aims, a staged approach was undertaken and the system development lifecycle was used as a structure for describing the stages of development and the iterative approach [40]. For this paper, we focus on the first 3 phases: analysis, design, and implementation.

### Analysis Phase

During the analysis phase, we wanted to inform the key components of the app through a search of the literature. We had already established that this type of app on smartphones did not exist and instead used the exercise and psychological literature to establish important components of the app.

We then conducted an informal consultation with a community-based strength and balance exercise class of older adults. The 12 older adults (10 women and 2 men) attending the class were asked for their opinions on using an app to set goals and home exercises and then how they would feel about receiving messages and prompts. We also asked how they would feel about reporting their exercises on the phone. Furthermore, the older adults were asked to share examples of the types of outcome-based goals they might set.

Subsequently, we approached exercise instructors delivering the evidence-based program through Later Life Training (LLT). LLT is the largest provider of training to deliver the FaME and Otago program in the United Kingdom [41]. This was done through their Facebook page. We asked instructors what types of goals they set with older adults and the feedback that they gave them during their programs. We also approached 3 fall services (9 physiotherapists, 2 rehabilitation assistants, 2 occupational therapists, and 3 nurses). We asked them what they thought about the initial concept of the app. This included using pictures from exercise booklets provided by LLT, as these are commonly used by falls services in the United Kingdom, examples of goals they could set, and feedback given to patients.

### Design Phase

During the second design phase, HH (health care researcher) and CT and SM (computer scientist and engineer, respectively) worked together to put together 2 basic prototype apps based on wireframes. This started by creating diagrams of how the app would flow and the functions, then there were further discussions, and then the creation of the prototypes. As part of this phase, we also looked at the types of BCTs that we had found based on the literature and discussions and mapped them to the Behavior Change Taxonomy by Michie et al [33]. These are reported in detail in our trial protocol paper [42].

Once the initial prototypes were created, 2 patient and public involvement (PPI) app development workshops were conducted to gain initial feedback on the concept (perceived usefulness), basic design (ease of use), and approach developed so far with the following groups:

1. Group of health care professionals (n=5) from a Manchester Falls Service (2 physiotherapists, 1 occupational therapist, 1 rehabilitation assistant, and 1 assistant practitioner).
2. Group of older adults were aged 60 years and above (n=8), community dwelling, and independent living from an age UK strength and balance falls exercise group.

The health care professional workshop was run by a researcher who was also an occupational therapist (OT) in a different fall team. The older adult workshop was run by the OT and lead researcher for the project. In the workshops, the initial concept of the technology was discussed with an explanation of why we thought it was important (perceived usefulness), what we were trying to achieve, and how the apps would work. We connected the phone to a large screen and demonstrated to the group what patients would have to do with the app. For health care professionals, we demonstrated what they and patients would have to do. This was followed by giving participants the opportunity to use the apps themselves. We had several phones, and participants in both workshops took turns to navigate around the apps. The older adults could see examples of exercise programs that had been scheduled and have a go at reporting their exercises. Health care professionals tried goal setting. We discussed participants’ thoughts on a one-on-one basis as they tested the apps and then brought everyone back together for further discussion. At this point, feedback messages had not yet been programmed, but potential examples were discussed. Notes were not only taken on the feedback provided but also on observations of participants’ use of the phones.

Contact with older adults and health care professionals in these first 2 phases of development was classified as patient and participant involvement (PPI). Therefore, we only collected aggregate details on gender, ethnicity, previous experience of smartphone or tablet use for older adults and gender and clinical background for health care professionals.

### Implementation Phase

#### Usability Study

This stage was used to determine the acceptability and usability of the apps with patients and health care professionals and included participatory evaluation, testing, interviews, and focus groups. The usability study ran for 3 weeks and tested the acceptability and usability of the technology as part of the exercise intervention.

The research proposed in this stage is predominantly qualitative but forms part of a larger mixed methods approach [43]. This approach enables us to establish whether the technology is acceptable to patients and health care professionals (qualitative methods) and assess its usability (technology testing), making improvements if required. The study was granted ethical approval by the North West Greater Manchester Central NHS Ethics Committee. The usability study has allowed for the planning of a subsequent feasibility randomized controlled trial and the design of the apps [42].

##### Sampling Principles and Procedures

Patients at risk of falls (aged 50 years and older), identified through one community fall rehabilitation service in Manchester, were recruited. As this was a pragmatic study, participants were those who would usually be offered a home exercise program by the service and could be at any stage in their rehabilitation. Patients who were unable to follow instructions were excluded, as were those with severe visual or hearing impairment. At this point, there were no other exclusion criteria. The first 20 eligible patients who were currently attending the service who were willing to participate were recruited. A total of 3 health care professionals gave patients the study information sheet and informed them about the technology. The apps were demonstrated to patients before they were asked to give informed consent. All patients recruited were offered a one-on-one interview in their own home. If they had a family member present, they could also join.

Health care professionals from 3 fall services in Manchester were recruited to participate in 3 focus groups. All members of staff (n=17) in each team were given study information by their team leader and asked if they were available for a focus group, which then took place at their place of work.

##### The Intervention

For the testing, we used the Samsung Galaxy S4 with pay-as-you-go SIM cards and 4G, and where possible, we connected them to the patients’ Wi-Fi. The apps tested on the smartphone included the following: (1) “Motivate Me” app: used by the health care professional in consultation with the patient to set outcome and behavior goals, including scheduling their exercises. The health care professional can then view the patients’ exercise reports sent from “My Activity Programme” and send feedback messages. “Motivate Me” sends the patients’ exercise program to their “My Activity Programme” app. (2) “My Activity Programme” used by the patient to view their exercise program sent through Motivate Me reports their exercises back to the health care professional and receives prompts to exercise and motivational messages as pop-ups (Multimedia Appendix 1).

The exercise program, goal setting, and feedback were delivered by a health care professional. The health care professional delivered the Otago [7] and FaME [8] exercises. These were delivered once a week face-to-face with a health care professional in the patients’ home and they were encouraged to exercise at least three times a week. Exercises delivered on “My Activity Programme” were adapted and tailored to the patient’s individual needs based on a pre-exercise assessment already conducted by the service and through goal setting on the app. All patients were given a home exercise booklet, which is part of the standard service.

##### Testing Procedures and Measurements

Patients were linked to one health care professional to support them with their program and carry out the intervention. This was allocated as would be in standard practice (not influenced by the research team). Issues the health care professional (deliverer) and the patient (recipient) had with the smartphone technology throughout the testing period were recorded (issue log and field notes). We recorded all issues but were particularly focused on perceived usefulness (requirement for internet access or testing of 4G through mobile phone, whether patients received messages) and ease of use (use of touch screen for reporting, ease or acceptability of reporting, whether health care professionals could use the phone to set goals) [37].

##### Interviews and Focus Groups

Following the testing period, health care professionals from 3 services delivering fall rehabilitation were recruited to participate in 3 focus groups. The service involved in the testing gave direct feedback on their experiences of using the technology. The other 2 services received a demonstration of the technology and were asked to give their feedback based on a similar interview schedule.

Patients who participated took part in a one-on-one interview in their own home. The interview and focus group schedules were based on the FAll Repository for the design of Smart and sElf-adaptive Environments prolonging Independent livinG (FARSEEING) [44] consortium guidelines (Multimedia Appendix 2). Participants demonstrated their use of the phone to the researcher. Key areas were explored in relation to the “Motivate Me” and “My Activity Programme” and can be mapped to either ease of use or perceived usefulness.

#### Data Analysis

Data from the issue logs were collated and summarized, and comments were added to the qualitative data analysis. Data from the issue logs provided triangulation for focus group or interview data.

Follow-up interviews with patients, focus group data with health care professionals, and field notes were analyzed together using a framework analysis, where the questions around the different apps provided a natural structure for the coding [45]. The NVivo 11 (QSR International) qualitative data analysis software was used to manage the data. The validity of the analysis was checked by returning to the data once themes were identified and through the use of a second researcher who carried out independent coding. Codes that emerged were discussed between 2 researchers, which is an approach that ensures rigor [46].

## Results

### Analysis Phase

The initial motivation for the development of the apps came from the literature examining previous studies looking at exercise adherence [13,18,25,26,28,30,32]. We identified the importance of goal setting and setting outcome-based goals (what is it that patients would like to do that they cannot do now) [25,30,32]. We also identified the value of feedback as a mechanism for building self-efficacy and as a means of social support in the exercise and mobile health interventions [14,15,18,32,47].

Health care professionals across the 3 fall services told us that they already carried out goal setting with patients on the first assessment but that this was informal. They indicated that an app that would support this process in a formalized way and providing feedback to patients would enhance current practice.

On the basis of the literature [25,29,30,32] and the discussion with health care professionals, we decided to develop long-term outcome-based goals as an important part of the app. Health care professionals usually ask patients about long-term outcome-based goals at the point of first assessment, so we wanted the app to facilitate this. Within the app, we offered a range of preset goals and based on feedback from health care professionals, grouped them into physical (physical ability or task specific) and emotional goals (I want to feel more confident).

The literature suggests that personalization of the exercise program and technology is important to patients [21] and that older adults prefer limited interaction with technology [21]. To ensure that the app was personalized to the individual and responsive to their needs and requirements, we decided to ask health care professionals to do most of the personalization. Therefore, 2 apps were created; one for the health care professional (to carry out goal setting with the patient and to communicate with them) and one for the patient (to receive messages and view exercises or goals). The health care professional could then guide the patient through the goal-setting process and personalize their goals and program. The patients’ goals and program would then transfer from their phone to the patients’ phone, requiring less interaction and less technical ability from the patient. As sensors in the phone are still unreliable in detecting static exercises without constantly moving the position of the phone or additional sensors [48], we decided not to use automatic detection of exercise through the phone but rather to include self-reporting of exercises within the patient app, which the health care professional could then see. This information provides an indication of the patient’s progress and adherence.

**Figure 1:**
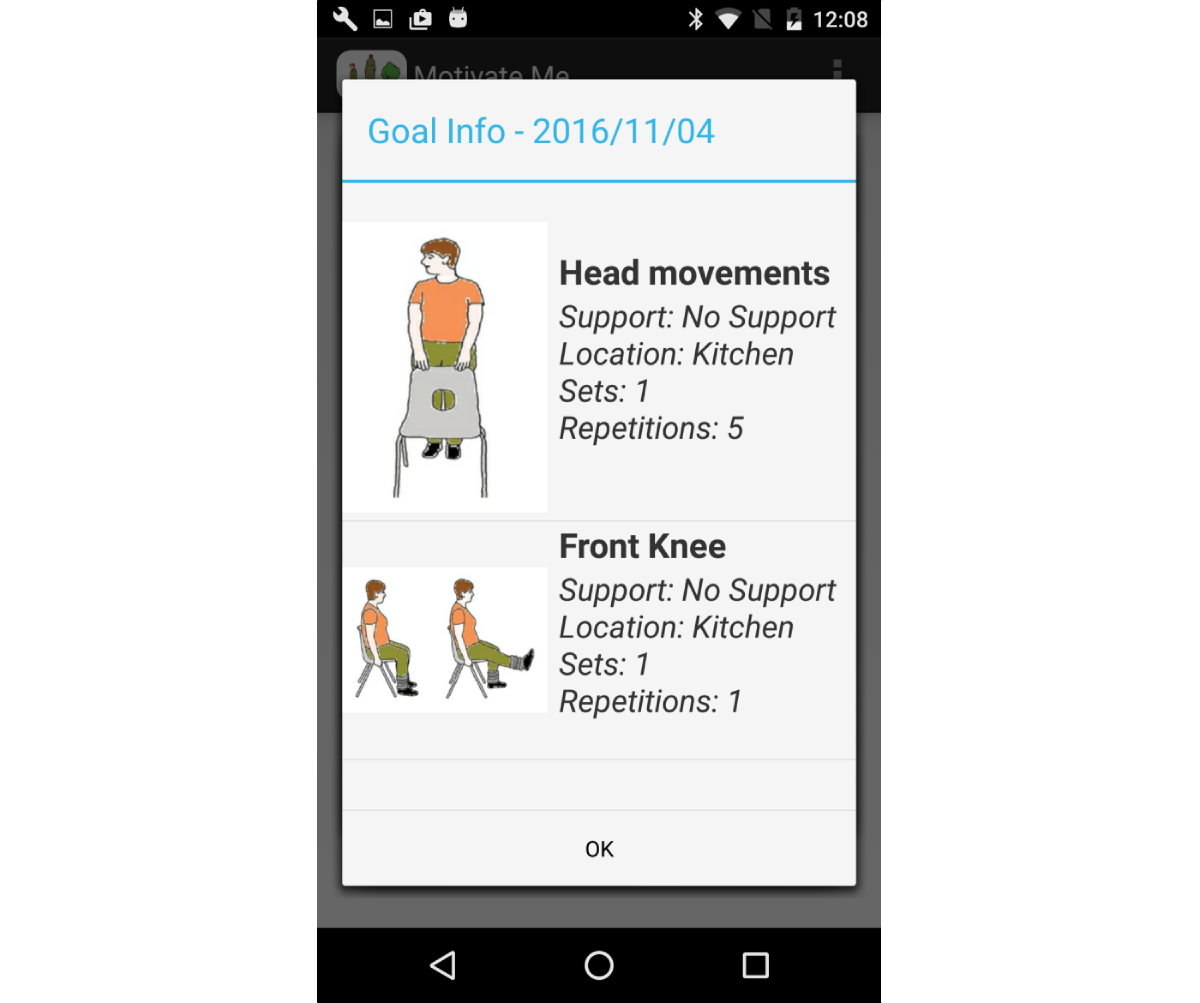
Interface: example of exercises.

We decided to use pictures of the exercises included in the LLT’s home exercise booklets (Figure 1). Health care professionals liked the idea of using icons that they and patients were familiar with. We decided to adopt the name “Motivate Me” for the health care professional app. “Motivate Me” is LLT’s motivational training for working with older adults and the most adopted motivational training across the United Kingdom [41]. We adopted the name “My Activity Programme” for the patient app because health care professional and older adult feedback suggested we avoided the word “exercise.”

BCTs proposed as part of the apps, based on this initial consultation and the evidence [33], include goal setting (behavior and outcome), action planning (recording the plan to exercise in a diary on the smartphone or reminder text messages when it is time to start the program), and feedback on behavior (providing feedback on what they have done or benefits).

### Design Phase

#### Basic Prototype

Following this initial consultation and development work that established the potential scope and requirements for the app, we created storyboards and app flow or options for the operationalization of the apps. Prototypes were then created.

In the initial prototypes, both apps had a very simple design, including pale yellow buttons, black writing, and large font to aid visual impairment. No messages were displayed in the initial prototype.

#### PPI Workshops

##### Older Adults Workshop

Demographics of participants in both workshops are reported in Multimedia Appendix 3. We observed when we first introduced the concept of the technology that the majority of the older adults (6/8, 75%) were reticent about using smartphones, describing them as frightening. The two who were comfortable with the concept were the participants who had previously used a smartphone or tablet. However, it was observed by the facilitators later on in the group discussion that the older adults having held the phone, looked at the app and found that they were able to use it had some of their fears allayed (6/8, 75% were then positive about using the app). This supports the idea that involving users in the development of new technology means they are more likely to adopt it [21]. Further details of the feedback are outlined in Multimedia Appendix 4. Following these initial workshops, we created “How to” guides for health care professionals and older adults liaising with patients on our study advisory board.

### Implementation Phase

In total, 7 patients (4 men; mean age 77.1, SD 8.53 years; range 64-92) took part, 6 agreed to be interviewed, and for one interview, the patient’s son was also present. Only two of the patients who took part already owned a smartphone. Further demographics are reported in Multimedia Appendix 1. During the testing, 2 physiotherapists (2 patients each) and 1 occupational therapist (3 patients) used “Motivate Me.” In total, 11 health care professionals participated in the focus groups.

Data from the issue log are summarized in Table 1, with relatively few issues with the apps. We asked patients and health care professionals whether they used the “How to guides.” Patients and health care professionals did not feel that they needed to use the guides.

Data were summarized under the two different smartphone apps and then the themes, barriers, facilitators, and building functionality, with 9 further subthemes. There were 2 additional cross-cutting themes: phone usability and confidence. First, we present the cross-cutting themes and then individual themes (Multimedia Appendix 5). Some themes only occurred in health care professional or patient data.

**Table 1:** Issue log data.

Participant and issue		Number of occurrences	Changes made to app
**Patients**			
	Delay in sync between the health care professional’s phone and patient’s phone	1	Fast sync capability added
	Patients missing messages	2	Pop-up stays on phone until patient says “ok” or “I like it”
**Health care professionals**			
	Amending exercises time consuming on health care professional’s phone	1	Process streamlined
	Setting goals because of new year. 2017 options not available	1	Glitch in system amended immediately

#### Cross-Cutting Themes

##### Phone Usability

Two patients found phone usability difficult in relation to the touch screen. Both had arthritis in their fingers and difficulty using the phone, and one patient was still able to use the phone with practice. The other patient decided that she did not like smartphones and stopped using it altogether. Others had no difficulties using it, even those who had never used a smartphone before. One patient wanted to keep the phone at the end of the study and asked the team further details about purchasing one. Two patients already had a Samsung phone and liked the familiarity of the phone. Patients and health care professionals commented on the standard messages sent by the network provider being annoying.

#### Confidence in Technology

Having confidence to use different technologies was an important factor in whether they were adopted for both health care professionals and patients. Health care professionals had a fear that the technology would put some patients off their rehabilitation program. Not all of the health care professionals supporting the usability testing were very technology minded and thus felt that there was a learning curve. However, it was observed that showing confidence was important for patients for them to have confidence. There was a view from health care professionals and patients that, as older adults used phones generally in life, confidence would increase.

For patients, there was confidence to be able to use the technology in the first place, and some patients regardless of support did not really engage with the technology. All of the patients who tried using the phone increased their confidence and ability. Once patients had built their confidence, they could then utilize the smartphone for other purposes.

Family support was important in building patients’ confidence, whether this was by children or spouses. Health care professionals reported that most families would support patients to use the technology.

#### Barriers

##### Types and Delivery of Messages

Patients at times missed motivational messages and prompts in “My Activity programme.” Messages were delivered as pop-ups in the app, with no sound given, and they stayed on the screen for 5 min. For those with an existing phone, this was particularly the case because they were given a study phone rather than using the apps on their own phones. Patients received a daily message in the app asking about their health and giving them the option to suspend the messages if they were unwell, the format of this message caused particular confusion for one participant.

We asked patients about the potential of receiving voice messages rather than pop-up messages and whether they would find them more motivating. In general, patients felt that it could be intrusive. The family member of a patient said that if a health care professional was going to send voice messages, prerecorded or in real-time, they may as well ring them.

Health care professionals had mixed views about the potential for sending voice messages, and there was a concern that patients would not want to hear their voices. Health care professionals again spoke about the potential for these kinds of messages to be intrusive, unless in certain contexts. In the context of patients’ exercises, they had to be motivational as they felt that instructional messages related to exercises could cause risk if the health care professional could not see what they were doing.

##### Icons and Pictures

The pictures of the exercises including the use of a chair in “Motivate Me” and “My Activity Programme” were raised as an issue by health care professionals in all 3 services. This was also an issue in the home exercise booklets they handed out. Patients would follow the pictures rather than follow the therapists’ direct instructions. Health care professionals encourage patients to carry out their exercises in the kitchen using the worktop for support, rather than a chair. Patients did not comment on the icons or pictures.

##### App Flow

There were few changes suggested by participants for “My Activity Programme.” However, to enable the app to be simplified, there were suggested changes for “Motivate Me.” Health care professionals felt that the app was not easy enough to use and could be made more intuitive, particularly when setting goals with patients. There was an issue with the app fitting in their way of practice. Some services first ask patients what they want to achieve and pick the exercises related to those outcome-based goals. However, the team we worked with said that because it was an evidence-based program, they deliver all of the exercises as long as appropriate and tailor the feedback they gave to the patient dependent on their goal.

There was a suggestion that setting both types of goals (outcome and behavioral) in one process led to errors and a risk of work loss if a mistake was made. They suggested that the process needed to be broken up into 2 stages.

#### Facilitators

##### The Apps as a Communication Tool

###### My Activity Programme

Health care professionals were initially worried about us asking patients to report individual exercises, suggesting that it might be too demanding. We decided to test how much they would be willing to report with the option of reducing this if feedback was negative. Patients were happy to report their exercises and found it a satisfying way of communicating with their health care professional. They also liked to receive messages and found them helpful, although some would have liked to be able to respond to messages.

###### Motivate Me

Health care professionals saw their app as a good way to communicate with patients and see what they had reported. If the patient had not reported their exercises, then the health care professional could use the app to provide encouragement. They could also use it as a communication tool to support patients when they are not with them. It was not only seen as a communication tool in the short term during regular contact but also for long-term follow-up of patients after discharge.

##### Good App Usability

Patients reported good usability for “My Activity Programme” telling health care professionals during the testing that it was easy to use. There were very few suggested changes. Patients also reported that they could view their exercises on the app with no issues, even those who did not like using the smartphone managed to navigate the app.

##### Goal-Setting Functions

Goal setting is an important part of the behavioral intervention within “Motivate Me.” A part of usability was that health care professionals felt that it fit within their existing practice. Patients also liked the idea of goal setting together with the health care professional using the app, feeling that their needs and expectations were being considered.

##### Flexibility of Use

Health care professionals discussed the flexibility of the smartphone apps and how they could be used with different populations and services. They thought they could be used as preventative apps for exercise instructors and charitable organizations who may be delivering evidence-based fall prevention exercises. They also felt that they could be important tools for other at-risk populations.

#### Building Functionality

##### More Flexibility in Times

In terms of the functions in “Motivate Me,” there were some changes that patients asked for when setting goals. They did not always want to specify a time when they were going to do home exercise; instead, they wanted to fit it around their daily lives. We asked them whether they were happy to schedule a day to exercise, and they preferred to set specific days without specific times. Patients were happy to schedule a time when they were exercising with the health care professional, so flexibility was required within the app.

##### Additional Information

###### Motivate Me

Health care professionals thought it would be helpful for patients if they could add notes underneath where they had set the exercises, as they did in home exercise booklets. They discussed how it would be useful to use a keyword to search for goals within the library.

###### My Activity Programme

Health care professionals and patients suggested that patients should be able to view their goals (and exercises) at any point. In the prototype, patients could only view them on the days they had set to exercise, and it was felt that this caused inflexibility and confusion. Health care professionals wanted to check the patients’ phone so they could see that the goals had gone into their app, in the current format they could not do this.

Patients and health care professionals talked about whether the motivational messages could stay on the screen for longer so they did not get missed. Some patients requested a loud noise when the pop-up came through to prevent them from missing it. Health care professionals also talked about not knowing whether patients had seen the messages and whether we could ask patients to say they “liked them,” and then see this in their app.

Finally, one service discussed whether “My Activity Programme” could include videos of the exercises. There was a discussion about patients sending health care professionals’ video clips of them carrying out the exercises. However, if this was too complex, then health care professionals discussed the potential to at least link the app to other resources, existing systems, and websites they already had in place where patients could access videos.

## Discussion

### Principal Findings

Introducing technology to patients and health care professionals is challenging, with a range of barriers around usability and acceptability. However, although barriers to the use of the apps were identified within this study, solutions were also offered by patients and health care professionals. Getting users involved in the development of technologies is recommended as key to their success, and we think this has led to very few changes suggested to the “My Activity Programme” during the implementation phase [21]. The iterative approach used through HCD has been found to be successful in creating apps that are usable for older adults and clinicians [34,36] and has enabled us to ensure that the apps are flexible and can continue to evolve. Our app development fits within a user-led design approach and we have consulted older adults, patients, and health care professionals not only about their needs and at key design points but also throughout the design process [49]. We liaised with different sets of health care professionals and both older adults in the community, previous patients of fall services, and current patients through the different stages of development to enable diverse inputs. Overall, we found that creating two apps and making “My Activity Programme” simple, tailored, and personalized helped to make it more acceptable to patients and alleviated fears of technology.

Results from the analysis phase indicated that goal setting (outcomes and behavior) and feedback would enhance current practice. Collecting example goals from both health care professionals, instructors, and older adults was an important part of ensuring that the apps could be personalized, which is essential [50]. Using recognizable and relevant goals and icons for the exercises were thought to make professionals and patients feel more confident.

The results of the implementation phase reflected earlier findings from the analysis phase, suggesting that there were initial barriers to phone use. Issues with the touch screen were discussed as a barrier to use, particularly where patients had arthritis and poor finger dexterity. The TAM focuses on perceived ease of use and the role it plays in acceptance [37]. A smartphone is needed for these types of apps, and from previous research, we know that patients would prefer not to navigate new technology [14]. All current smartphones are provided with a touch screen, and further work is being carried out to explore their use [51]. However, it was suggested that the use of a smartphone pen would overcome most of the issues. Text messages from network providers were perceived by health care professionals as annoying, the option to use roaming SIM cards (across networks) to increase 3G/4G coverage now means that this does not have to be an issue.

Ensuring that the apps fit the needs of health care professionals and patients was an important part of our development process. Confidence in using the technology is important for health care professionals and patients. Health care professionals are more likely to adopt technological interventions if they perceive the benefit to themselves and patients [52]. The TAM focuses on the acceptance of technology being related to its perceived usefulness [37]. Health care professionals saw their confidence as important in encouraging patient confidence in the use of the technology. There was a perception from health care professionals and patients that the more they used the technology the more confident they would become, something established in previous research [52,53]. The support of family and friends played an important role in supporting patients’ attitudes toward technology and confidence to use it [21].

Feedback on the apps provides further insight into the potentially acceptable delivery of messages. We found that pop-up messages were mostly acceptable and a key communication tool between health care professionals and patients. Personalized and tailored messages directly from the health care professional garnered the best response in our testing, and a recent evidence review supports this [54]. There is currently no clear evidence on what older adults find motivating in terms of number, type, and delivery of messages from an app that supports fall rehabilitation. We know that to encourage participation in fall prevention technologies, we should emphasize the benefits of positive active aging [21] and that messages should be positively framed [44,55].

For the apps to be acceptable, they had to fit with health care professionals’ practice as well as be flexible and fit with older adults’ needs. Previous research suggests that technological solutions for fall prevention need to be individually tailored [21]. Interviews with patients suggested flexibility around goal setting and message delivery so that the use of the technology was not restrictive and fit with their lives. Focus groups with staff-provided suggestions of how “Motivate Me” could be improved so that further individualization was offered, for example, adding notes and ensuring the process of goal setting more closely reflected practice. Health care professionals discussed other additional features that could enhance the app, for example, videos, which need to be carefully considered alongside burden on the participant and data confidentiality, issues previously raised as barriers [21]. Health care professionals also identified the potential for the apps to be used with different populations and for follow-up after the rehabilitation phase.

“My Activity Programme” included asking patients to self-report their rehabilitation exercises. There is a lack of well-validated self-report measures for recording adherence in the specific context of prescribed but unsupervised home-based rehabilitation exercises for older adults [56]. Studies have found a poor correlation between self-reported exercise questionnaires and objective measures such as accelerometers [57,58]. However, Fukuoka et al [59] found good compliance and correlation between self-reported activity on an app and a step counter. Objective measurement of rehabilitation exercises is difficult without asking patients to wear multiple sensors [60], something that has shown promise but has usability issues, particularly with this patient cohort [21].

During the development phase, health care professionals questioned whether participants would be willing to report individual exercises, although health care professionals found information on type of exercise, dose, and intensity useful. We found that patients were happy to report their exercises and, at times, found this motivation, which is related to the general literature on self-monitoring [61]. This app could in fact provide important contextual data for those trying to monitor people’s physical activity as the sensors in the phone alone are currently inadequate at detecting static strength and balance [60].

### Limitations

There were limitations to the study. As part of the analysis phase of the study, we decided to move to a system that used 2 apps that interacted (setting goals on “Motivate Me” and using “My Activity Programme” to receive messages and report exercises). This approach mirrored practices as the health care professional will set the patients’ exercise program in consultation with them. It also made the patient app easier to use. We did not ask older adults in the workshop or patients in the interviews directly about whether they would like to be able to change their exercise schedule or goals themselves, but instead asked open-ended questions about the apps. This never arose as an issue for either older adults in the workshop or patients in interviews. However, it may be that some patients could find that the inability to change their program themselves (eg, when they planned to exercise and their outcome goal) negatively impacts their motivation; we know that control is important to use [21]. We need to balance functionality with the capabilities of our target group, and this is an important consideration for future iterations and something that may occur with longer use. There were very few changes to “My Activity Programme” suggested by older adults in the workshop. This could be because at this initial stage, not all of the functionality was in place (eg, messages did not come through at this point); therefore, they mostly focused on its look and style. Apart from explaining how the goal setting would work with health care professionals and asking for feedback on ideas for goals and messages, we did not show older adults “Motivate Me” in the first workshop. This was because we primarily wanted feedback from health care professionals at this stage and did not want to overwhelm older adults with something they would not directly use. However, further data from older adults could have proved useful.

The workshop was carried out with community-dwelling older adults who attended a strength and balance class, whereas the testing was carried out directly with patients. It could be argued that they were two different populations, and this may have influenced feedback. We argue that it is a strength to represent a wide variety of older adults’ views. We have a good representation of men and women in terms of older adults, patients, and health care professionals. Although usability testing was performed with patients aged 50 years and older, we wanted to test the app directly with the population who would use it. Recruitment took longer than anticipated and was exacerbated by the Christmas period. Therefore, a much smaller number of patients were recruited than planned. There was a lack of recruitment from Black and minority ethnic populations. However, the participants recruited represented a good mix of patients in terms of comorbidities, age, gender, and prior experience with technology. The period we tested the technology over was relatively short and may not have identified all implementation and usability issues. We also could have used a usability questionnaire. However, at this point, this was a predominantly qualitative initial development, acceptability, and usability work designed to lead to further feasibility work.

### Conclusions

Overall, we have established that “Motivate Me” and “My Activity Programme” were acceptable to most older adults and patients who participated and all health care professionals. We developed important personalized content for our apps. There is a lack of research on smartphone-based interventions for the support of fall management and prevention. This study enabled us to build 2 apps that have the potential to support health care professionals and patients with their rehabilitation program. “Motivate Me” and “My Activity Programme” require further improvement and development and then need to be explored in practice as part of a bigger feasibility trial.

## Appendix

Multimedia Appendix 1 Functions of the applications at each stage of development.

Multimedia Appendix 2 Interview and focus group schedules.

Multimedia Appendix 3 Demographics of participants for workshops and usability study.

Multimedia Appendix 4 Feedback from workshops and corresponding changes made.

Multimedia Appendix 5 Themes from interviews and focus groups.

## Abbreviations

BCT: behavior change technique
FaME: falls management exercise
HCD: human-centered design
LLT: later life training
PPI: patient and public involvement
TAM: technology acceptance model
TPB: theory of planned behavior
SDT: self-determination theory
