# Supplementary material for: Smartphone Apps to Support Falls Rehabilitation Exercise: App Development and Usability and Acceptability Study
Source: JMIR Mhealth Uhealth. 2020 Sep 28;8(9):e15460. doi: 10.2196/15460 (PMC7551104; doi:10.2196/15460)
Supplement: Multimedia Appendix 1 [file mhealth_v8i9e15460_app1.docx]

**Multimedia Appendix 1:** Functions of the applications at each stage of development

| **Functions** | **Design Stage (workshops)** | **Implementation Stage (testing)** |
| --- | --- | --- |
|  |  |  |
| **Motivate Me** |  |  |
| Goal setting |  |  |
|  | Health professional can select a choice of pre-set outcome-based goals for patients.   Exercises from the Otago/FaME programme are pre-linked to the outcome goal.  Health professionals can select when the patient will exercise (days/times) | Health professional can select a choice of pre-set outcome-based goals for patients.  They can create a personalised free text goal  Health professional can select any exercises from the Otago/FaME programme and link them to the outcome goal.  Health professionals can amend the exercise and dates of exercise in an existing goal. |
| Messages |  | Health professional can send personalised messages to the patient. |
| Feedback |  | Health professional can view the exercise the patient has reported. |
| **My Activity Programme** |  |  |
| Personalised programme | Older people could see an example of a personalised exercise programme which has been set through Motivate Me. | Patients can see their own personalised exercise programme in the app which has been set through Motivate Me. |
| Reporting exercises | They could report exercises through the app. | They could report exercises through the app. Including reps/sets/minutes/seconds/weights/bands |
| Reporting health |  | Patients could suspend messages if they were not very well. |
| Receive messages. |  | Patients receive messages as pop-ups including prompts to exercise, messages related to their outcome goal (s) and personal messages from the healthcare professional. |
