# Supplementary material for: Smartphone Apps to Support Falls Rehabilitation Exercise: App Development and Usability and Acceptability Study
Source: JMIR Mhealth Uhealth. 2020 Sep 28;8(9):e15460. doi: 10.2196/15460 (PMC7551104; doi:10.2196/15460)
Supplement: Multimedia Appendix 2 [file mhealth_v8i9e15460_app2.docx]

**Multimedia Appendix 2:** Interview and focus group schedules

| **Patient questions** | **Farseeing guideline** | **TAM** |
| --- | --- | --- |
|  |  |  |
| **1. What did you like or dislike about using the smartphone?** |  |  |
| What did you think about wearing it in your pocket? | Ease of use and  adapted products. Comfortable to wear | Perceived ease of use |
| What did you think about wearing it around  your waist?^a^ | Ease of use and  adapted products. Comfortable to wear | Perceived ease of use |
| What did you think about the screen size and  what you could see on the smartphone? | Ease of use and adapted products. Clear screens | Perceived ease of use |
| What did you think about the touch screen  and navigating your way around the smartphone? | Ease of use and  adapted products. Clear screens | Perceived ease of use |
| Did you feel that you had enough support to  be able to use the smartphone? | Ease of use and  adapted products. Demonstrate use | Perceived ease of use |
| Did you feel like it fit in with your lifestyle?  How did you feel about wearing it/carrying it  around? | Ease of use and adapted products. Home and Lifestyle | Perceived ease of use |
| Did you feel that family and friends were  supportive of you using the smartphone and did this influence how you used it? | Home and Lifestyle Choice and control | Perceived ease of use Perceived usefulness Subjective norms |
| **What did you like or dislike about using the smartphone to record your exercise sessions?** |  |  |
| How easy was it to record your exercise sessions  on the smartphone? | Ease of use | Perceived ease of use |
| Could the way that you recorded your exercises be improved? | Ease of use | Perceived ease of use |
| How often were you happy to record your exercises? | Perceived need,  Convenience,  Preventing falls, being challenged | Perceived usefulness |
| **What did you like or dislike about the messages  you received on the smartphone?** |  |  |
| What did you think about the set-up of the phone  with your health professional at the start of the study (so that you could receive the messages)? | Choice and control, perceived need, being challenged, preventing  falls, independence. | Perceived usefulness  and perceived ease of  use. |
| Did the messages motivate you to keep on carrying  out your exercise sessions? | Perceived needs, being challenged, preventing  falls. | Perceived usefulness |
| Were you happy with when you received the  messages or would you have liked to receive them  more or less often? | Perceived need, being challenged, choice and control | Perceived usefulness  and perceived ease of  use. |
| Did you like or dislike being reminded to exercise? | Perceived need, being challenged, choice and control | Perceived usefulness and perceived ease of use. |
| **Overall** |  |  |
| Would you use the smartphone again or continue to  use it if you could? | Perceived need, ease  of use. | Perceived usefulness and perceived ease of  use. |
| If not, why not and which parts of using the  smartphone did you not like? | Perceived need, ease of  use. | Perceived usefulness  and perceived ease of  use. |
| What changes could we make for you to be happy  to use the smartphone in the future? | Perceived need, ease of  use. | Perceived usefulness  and perceived ease  of use. |
| **Health professional questions^b^** **ALL SERVICES: What do you like or dislike  about using the smartphone?** |  |  |
| CFS1: Did you feel that you had enough training  and support to use it with your patients? | Ease of use,  demonstrate use | Perceived ease of use. |
| CFS1: What did patients think about the  smartphone? | Perceived need, ease  of use. | Perceived usefulness  and perceived ease of  use. |
| **ALL: What did you like or dislike about being  able to set when patients would do their exercises on the smartphone?** |  |  |
| CFS: Did you think it was easy to set up on the  mobile phone when the patient would exercise? | Ease of use | Perceived usefulness  and perceived ease  of use. |
| ALL: What do you think about being able to  change when the patients will exercise remotely? | Perceived need, being challenged, preventing  falls, convenience | Perceived usefulness |
| ALL: How easy do you think it is for patients to  report when they have carried out their exercises? | Ease of use | Perceived ease of use. |
| ALL: Is there anything we can do to improve  how patients record their exercises? | Ease of use | Perceived ease of use. |
| **ALL: What did you like or dislike about the motivational messages on the smartphone?** |  |  |
| ALL: What do you think about setting goals on the smartphone? | Choice and control, perceived need, being challenged, preventing  falls, independence. | Perceived usefulness  and perceived ease  of use. |
| CFS: What were your experiences of setting goals with patients using the smartphone? | Choice and control, perceived need, being challenged, preventing  falls, independence. | Perceived usefulness  and perceived ease  of use. |
| ALL: What do you think about the messages patients can receive/did receive? | Choice and control, perceived need, being challenged, preventing  falls, independence. | Perceived usefulness |
| ALL: Are there any amendments/suggestions  you can make to the content/timing/visual  representation of the messages? | Choice and control, perceived need, being challenged, preventing  falls, independence. | Perceived usefulness  and perceived ease  of use. |
| ALL: Are there any improvements you can suggest to the motivational messages on the smartphone? | Choice and control, perceived need, being challenged, preventing falls, independence. | Perceived usefulness  and perceived ease  of use. |
| **Would you use the smartphone  again or continue to use it if you  could?** |  |  |
| If not, why not and which parts of using the  smartphone did you not like? | Perceived need, ease  of use. | Perceived usefulness  and perceived ease  of use. |
| What needs to be improved for using the system  in your routine practice? | Perceived need, ease  of use. | Perceived usefulness  and perceived ease of  use. |

^a^They were also testing it as a falls alarm.
^b^Some questions were only asked of the service related directly to app usage.
