# Supplementary material for: Smartphone Apps to Support Falls Rehabilitation Exercise: App Development and Usability and Acceptability Study
Source: JMIR Mhealth Uhealth. 2020 Sep 28;8(9):e15460. doi: 10.2196/15460 (PMC7551104; doi:10.2196/15460)
Supplement: Multimedia Appendix 3 [file mhealth_v8i9e15460_app3.docx]

Multimedia Appendix 3: Demographics of participants for workshops and usability study

|  | **PPI Workshops (Design)** | **Usability (Implementation)** |
| --- | --- | --- |
|  |  |  |
| **Older adults/Patients** | **N=8** | **N=7** |
| Gender | Female 6 | Men 4 |
| Age | Over 60 8 | Mean age 77.1 (SD 8.53, range 64-92) |
| Ethnicity | White British 8 | White British 4 |
|  |  | White Irish 2 |
|  |  | Indian 1 |
| Co-morbidities | Unknown 8 | 3 |
| Previous smartphone/tablet use | 2 | 2 |
| **Health Professionals** | **N=5** | **N=11** |
| Gender | Female 4 | Female 8 |
| Professional background |  |  |
|  | Physiotherapist 2 | Physiotherapist 9 |
|  | Nurse 0 | Nurse 1 |
|  | Occupational Therapist 1 | Occupational Therapist 1 |
|  | Rehabilitation assistant 2 | Rehabilitation assistant 0 |
| Ethnicity | White British **5** | White British 11 |
