# Supplementary material for: Smartphone Apps to Support Falls Rehabilitation Exercise: App Development and Usability and Acceptability Study
Source: JMIR Mhealth Uhealth. 2020 Sep 28;8(9):e15460. doi: 10.2196/15460 (PMC7551104; doi:10.2196/15460)
Supplement: Multimedia Appendix 4 [file mhealth_v8i9e15460_app4.docx]

**Multimedia Appendix 4.** Feedback from workshops and corresponding changes made.

| App function | Health care professionals | Older adults | Changes made |
| --- | --- | --- | --- |
| Motivate Me: goal setting (outcome-based goals) | - Professionals liked the idea of being able to pick from a “message library” - They would like to be able to go to a goal and amend it later on | Mostly happy with wording of preset goals   - Personal individualized goals preferred, linked to own lifestyle and health - Some of the goals did not feel relevant to the older people, for example one which stated—“to be able to play with grandchildren,” where the person in question didn’t have any grandchildren - They wanted goals about staying independent and not being reliant on others | Allowed the creation of personalized tailored goals if a suitable pre-set goal was not available  Additional goals that older adults felt were relevant were collected and included in the next version of the apps |
| Motivate Me: exercise prescription (behavioral goals) | - They want to be able to add more details for tailoring the exercises like reps and the location where the person should exercise using a drop-down list - They would like to have the full library of evidence-based exercises available to select from, so the professional could select the exercises themselves for the individual, rather than having them pre-linked to the preprogrammed goal - A drop-down list with hold on/not hold on was suggested for each exercise to increase safety - They would like to be able to select whether the person uses weights - They would like to be able to amend and add new exercises at a later date | - Did not discuss | - We added the capability to set the number of reps/seconds and weights/bands and location of where to do exercises - All exercises were made available for the health care professional to choose - Exercises could be updated and dates planned to exercise changed |
| Motivate Me: scheduling the exercise sessions | - Exercises are prescribed to be completed at least three times a week - They would like to be able to select from a list of days of the week - They would like flexibility in terms of length of the exercise program | - Did not discuss | - The flexibility requested was included in the app |
| Motivational messages | - Professionals were happy that these were selected from the library of messages as long as they were linked to the goals in some way - One professional thought that post exercise messages would be most motivating, like a reward for doing their exercises - They would like to select a number of messages to be sent at different times | - Patients were generally happy with the types of messages that might be sent and the prompts to exercise were seen as useful | - Health care professionals could select when to send messages and whether they would be sent prior or after the scheduled session |
| My Activity Programme (patient self-report app) | - Professionals thought that the reporting by patients would vary - One was concerned re: accuracy of feedback - One thought it would help with people with mild memory problems - Professionals felt that they would like to know how many exercises and reps the patient had achieved. They did express concerns around whether patients would be willing to report this much information - They requested that the word cancel was replaced by back | - Overall patients found it easy to report once they had tried it - No other comments made | - The word cancel was replaced by back - We decided to include as much information in the self-reporting part of the app as health care professionals would find useful and see if patients were willing to report it |
| Color, font, look of apps | - The professionals were in agreement with the older adults about the color and overall look of both apps | - Yellow for the background was selected as better for the visually impaired. No preference was stated for color or design of the apps; they were happy with them as they were | - The overall design of the apps was left as it was |
