# Supplementary material for: Smartphone Apps to Support Falls Rehabilitation Exercise: App Development and Usability and Acceptability Study
Source: JMIR Mhealth Uhealth. 2020 Sep 28;8(9):e15460. doi: 10.2196/15460 (PMC7551104; doi:10.2196/15460)
Supplement: Multimedia Appendix 5 [file mhealth_v8i9e15460_app5.docx]

**Multimedia Appendix 5.** Themes from interviews and focus groups.

| Theme and subtheme | | Quotes | |
| --- | --- | --- | --- |
|  | | Patients | Health care professionals |
|  | | | |
| **Cross-cutting themes** | | | |
|  | Phone usability | - “I was stroking the damn screen and nothing was happening and it wasn't until I'd used it for a couple of weeks that I was able to even use the touch screen” (Male, aged 82 years) - “I’m just not literate with these kinds of...I just can't deal with something like that…I think you did as much as you could really” (Male, aged 74 years) | - “There were lots of messages pop-ups. It didn’t bother me too much but it might bother the patients…” (Female, Occupational Therapist, S1) |

|  | Confidence in technology | - “Generally I think it’s good…my family and the children they accepted it” (Male, aged 82 years) | - “Because ultimately we don’t want to turn them off from the actual exercises… (Physiotherapist, female, S1)” - “I’ll just have to practise, because I’m not very good with technology (Physiotherapist, female, S3)” - “If we show confidence in the technology, they’ll feel more confident” (Physiotherapist, female, S1) - “I think it definitely has to be beneficial, and it won’t be proper for everybody, but for a lot of people it will be, and increasingly so as people become more confident with using technology” (Physiotherapist, female, S2) - “I was quite impressed about how they got used to them, the phones. I showed one patient how to use Google, one was taking photos” (Occupational Therapist, female, S1) - “…some families would be really pleased to be able to do that” (Physiotherapist, female, S1) |
| --- | --- | --- | --- |

| **Barriers** |
| --- |

|  | Types and delivery of messages | - “like today it stayed, but I don’t remember seeing that before” (Male, aged 82 years) - “It’s only like now and again where there’s something there, and I showed you, remember it was like in grey and I didn’t know whether that was yes or no” (Female, aged 69 years) - “If it just comes through whether you’ve got no choice, if it’s on a screen then okay but if it’s something you have to listen to it just feels a bit different...I don’t think I personally would find that beneficial…” (Male, aged 64 years) - “…it’s more work and more stuff you don’t need to do, you might as well just do a video call to the individual and speak to them that way…” (Family member of male aged 92 years) | - “I don’t think my voice is very motivational…oh, god, he’s on the phone again” (Physiotherapist, male, S3) - “That would be a problem I think…They do use that for people with cognitive impairment perhaps if they want to encourage…they record a family member who that person is familiar with…I suppose it could be nice, maybe motivational rather than instructional if it was an issue around them keeping up”. (Physiotherapist, female, S2) |
| --- | --- | --- | --- |
|  | Icons and pictures | —^a^ | - “If we can get rid of it, yeah, that’d be great, show something sturdy to hold onto, that’s what we prefer. Because that’s what we would go through in the precautions, and that’s the sort of thing that we write, in the kitchen, use the work surface. Because our pictures show a chair, and we’re not happy…” (Physiotherapist, female, S1) |
|  | App flow | — | - “I had a few problems about scheduling…I’d set up one, couldn’t schedule subsequent ones and had to come back out of it and try a different goal and managed to do it. So that was a bit frustrating. I think some of the goals could perhaps be simplified... so, if you like, little tweaks about that. (Occupational Therapist, female, S1) - “Because it’s an evidence-based programme. It’s the whole programme isn’t it? So I would rather extract some of the goals from what you’re giving rather than the other way round…it was quite clunky to do it that way” (Physiotherapist, female, S1) - “I’d had a phone call or something like that and I hadn’t pressed that button and that meant it didn’t upload…It is my mistake, but I just wonder if there is a way…” (Physiotherapist, female, S1) |

| **Facilitators** |
| --- |

|  | The apps as a communication tool | - “Because I was making an effort… I was doing it three times a week…I found it quite rewarding to record it some way ’cause I know I’d done it but it some ways it was letting somebody else know you’d done it...” (Male, aged 64 years) - “No, I would do that, I would follow that, that to me is help” (Male, aged 82 years) - “I’d prefer it two-way if I can have my say as well. I'd like it if XX said, no, you're not doing that right, you barmpot, I'd like to be able to say, get lost, or things like that” (Male, aged 82 years) | - “I’m just looking on the one here and you can see the feedback there and what they’ve done, which is quite good” (Physiotherapist, female, S1) - “If they’ve not given any feedback you could give them a message saying oh, I notice you’ve not fed back about your exercises, are you doing them” (Occupational Therapist, female, S1) - “Was very easy to use once you get in the routine of it. I think the routine’s the thing…I think it would be good because motivationally they’ve got something they can hold and they can press and they can physically see it. I think a black and white piece of paper with a picture on can be un-motivational sometimes. I think that’s it. And that’s what we’re going round doing, we’re kind of encouraged by visiting. Whereas this would be a constant encouragement everyday”. (Physiotherapist, Female, S2) - “What happens when they’re discharged…it could be very important” (Physiotherapist, Female, S2) |
| --- | --- | --- | --- |
|  | Good app usability | - “Yes, that was fine, showed me the exercises no problem” (Female, aged 81 years) - “course it’s easy enough” (Male, aged 92 years) | - “So that’s quite an indication isn’t it, if they’re saying actually it’s quite simple…that’s manageable” (Occupational Therapist, female, S1) |
|  | Goal-setting functions | - “I feel it’s good to be consulted on what you’re aiming for, rather than just being told that this is good for you. Yeah, I didn’t have any problems with that at all” (Male, aged 64 years) | - “It would fit in with what we already do, because we already discuss goals and set goals, and assessments” (Physiotherapist, male, S2) |
|  | Flexibility of use | — | - “We’re just doing work with Age UK at the moment…so they’re picking up those sort of people, so at that stage then the Age UK guy could use that” (Physiotherapist, female, S3) - “That sort of software you can use with anybody, it doesn’t have to be just fallers, it can be anyone with shoulder pains…to whatever really, it would work across the board” (Physiotherapist, female, S2) |

| **Building functionality** |
| --- |

|  | More flexibility in times | - “that is getting restrictive, I feel that I’ll do them when I want to do them... (Male, aged 82 years) - “It would be for me, personally. I need the opportunity, shall I say, because I've other things to do so I do them, whether it be in the morning or an afternoon or late in the evening” (Male, aged 64 years) | — |
| --- | --- | --- | --- |
|  | Additional information | - you know, something loud, then I know I’ve got a message…I’ve been missing the messages” (Female, Aged 69). | - “I also found that when you’re setting the exercises quite often, I’d give extra prompts or extra information…It’s something that we’ve mentioned before, but having extra space to be able to put…” (Occupational Therapist, female, S1) - “If it’s possible if there’s some way you can type a word in …balance and that it could bring up all the different goals that are to do with balance…” (Physiotherapist, female, S1) - “to be able to have a look back at what you’ve said would be really good…you couldn’t see it in their phone” (Occupational Therapist, female, S1) - “if there was almost like a returning method to say patient’s viewed that?” (Occupational Therapist, female, S1) - “it would be good if you could have some sort of video demonstration of the exercise…because obviously the diagram would do good, but if you had a visual there you could have someone, you could have a health professional speaking, telling them what to look for, and do like a 30-second clip. But then again I would imagine it’s the capacity of the app” (Physiotherapist, male, S2) - “you’ve got to rely on the trust of the patient to tell you; if you could do like a video diary… they’d record themselves doing a video, and then as a physio we could review them and make sure… because they could be doing their exercises, but they could be doing them wrong… It’s also going to take a lot of clinician time up”. (Physiotherapist, male, S3) - “we’ve got this new website up called, My Health My Community, and we’ve put loads of videos of the sort of more common exercise and stuff…there’s a video of it, so someone can play and go, ah, right, that’s what I’m doing. So maybe you could have a link on the app…” (Physiotherapist, male, S3) |

**^a^ – is used where the theme did not occur for the participants.**
